# Occurrence of acrylamide carcinogen in Arabic coffee *Qahwa*, coffee and tea from Saudi Arabian market

**DOI:** 10.1038/srep41995

**Published:** 2017-02-02

**Authors:** Mohammad Rizwan Khan, Zeid Abdullah Alothman, Mu Naushad, Ahmed Khodran Alomary, Sulaiman Mohammed Alfadul, Ibrahim Hotan Alsohaimi, Mohammad Saad Algamdi

**Affiliations:** 1 Department of Chemistry, College of Science, King Saud University, P.O. Box 2455, Riyadh 11451, Kingdom of Saudi Arabia; 2 King Abdulaziz City for Science and Technology, P.O. Box 6086, Riyadh 11442, Kingdom of Saudi Arabia

## Abstract

The present work describes the outcomes of the assessment on acrylamide contents in a number of thermally treated foods (Arabic coffee *Qahwa*, coffee and tea) obtained from the Saudi Arabian markets. A total of 56 food samples of different brands and origin were studied, the amounts of acrylamide in Arabic coffee *Qahwa*, coffee and tea were obtained in the range of 10 to 682 μg kg^−1^. In comparison to coffee (152–682 μg kg^−1^), the Arabic coffee *Qahwa* (73–108 μg kg^−1^) and tea (10–97 μg kg^−1^) contain lower amounts of acrylamide. Among the analyzed samples, the green tea contained low amounts of acrylamide ranged from 10 to 18 μg kg^−1^, and thus the green tea could be considered as a healthier hot drink. A great variation of acrylamide formation has been observed in these food products. This divergence may be due to the initial concentration of amino acids especially asparagines and reducing sugars in food products, in addition to roasting temperature and time, pH and water activity. The obtained data can also be used in epidemiological investigation to estimate the acrylamide exposure from nutritional survey.

In 2002, the University of Stockholm and the Swedish National Food Administration have jointly publicized that a number of food products thermally processed at elevated temperature contain fairly high amounts of acrylamide^1^. Since then, acrylamide gained a substantial awareness and it is known as process food contaminant^2^. The acrylamide is generated in the consequence of thermal treatment of foodstuffs via Maillard reaction, a reaction between the free amino acid mainly asparagines with reducing sugars at high cooking temperature^3^.

Previously, animal feeding investigation confirmed an association linking acrylamide in particular hemoglobin adducts and cooked animal foodstuffs^4^. To a certain extent, the long-term contact to acrylamide might cause harm to the nervous system including potential genetic and reproductive toxin in humans as well as in experimental animals^5^,^6^,^7^. In 1994, the International Agency for Research on Cancer (IARC) has classified acrylamide as ‘*probably carcinogenic to humans*’^8^. Recently, the European Food Safety Agency (EFSA) has proclaimed that the roasted coffee including other cooked foods for instance potatoes, crackers, cookies, toasted bread and breakfast cereals are the major source of acrylamide^9^, which contributes higher than 90% acrylamide intake through our daily diets^10^. The concentrations of acrylamide in some food products for instance potato chips and coffee reach up to 3500 μg kg^−1^ or even higher than 4500 μg kg^−1 ^11. These foodstuffs are significant from the opinion of contact estimation, either due to these foods holds a large amount of acrylamide compare to other food products or these food products are an element of the usual diet for the common inhabitants, therefore contain an elevated eating rates. Previously, the normal acrylamide ingestion through diet was described and found 1 μg kg^−1^ body weight day^−1^ and 4 μg kg^−1^ body weight day^−1^ for common inhabitants and higher consumers, respectively^9^,^10^.

In the recent years, the food lifestyles have significantly changed worldwide and gradually increasing eating of processed food products^12^. These food habits alteration are mainly apparent in juvenile populations whose lifestyles are allied with the consumption of high amounts of fast foods including Arabic coffee *Qahwa*, coffee and tea which supplies to the greater amounts of acrylamide ingestion^2^,^13^,^14^. In an attempt to assess the acrylamide exposure and threat assessment, widespread levels of information on acrylamide amounts in processed food products have been obtained. Up to now, a number of thousands information relating to acrylamide have been achieved and included in the US Food Drug Administration^15^ and European Commission Institute for Reference Materials and Measurements databases^16^. In the Saudi Arabian diets, thermally processed food products for instance Arabic coffee *Qahwa*, coffee and tea are very frequently present, and the Arabic coffee *Qahwa* is considered as a traditional hot drink. These food products are usually thermally processed in the form of roasting which usually favor the formation of acrylamide.

In the present study, to facilitate the entire assessment of acrylamide contents various Saudi Arabian food products especially Arabic coffee *Qahwa*, coffee and tea were examined. The achieved outcomes from the proposed investigation will increase the consciousness in the Saudi Arabian inhabitants together with the global population concerning acrylamide carcinogen, as well as it can be utilized to approximate the ingestion of acrylamide from such types of food products.

## Results and Discussion

Recently, the Joint FAO/WHO Expert Committee has described the explanatory statistics of acrylamide levels in various food products for the data collected between 2004 and 2009^17^. The average levels of acrylamide in main foods were: coffee (3–68 μg kg^−1^), potato chips (399–1202 μg kg^−1^), cookies (169–518 μg kg^−1^), French fries (159–963 μg kg^−1^), and crisp bread and crackers (87–459 μg kg^−1^)^17^. On the body weight basis, the average nutritional acrylamide ingestion for common inhabitants together with children, the acrylamide exposure in children are higher up to two folds relative to the adults population^17^. On the basis of earlier findings of acrylamide in thermally processed carbohydrate-rich foods and its exposure to the human beings, it is highly essential to perform the screening of toxic acrylamide in such type of food products frequently available in Saudi diets.

For the analysis of acrylamide, the performance of the method (quality parameters) in terms of linearity, limit of detection (LOD), limit of quantification (LOQ) and precisions (run–to–run and day–to–day) were established. The linearity of the method was determined in the concentration range of 8–2000 μg L^−1^ and excellent linearity (*R*^2^ = 0.999) was obtained. The LOD (signal-to-noise ratio, 3:1) and LOQ (signal-to-noise ratio, 10:1) were calculated and obtained 2 μg L^−1^ and 7 μg L^−1^, correspondingly. To establish the run–to–run precision, five replicates of a known amount of acrylamide standard solution (600 μg L^−1^) were analyzed on the same day. The relative standard deviation (RSD) was estimated from the five determined concentration levels, and the outcomes <2.0% were achieved. For the determination of day–to–day precision was determined by five replicates of same standard solution (600 μg L^−1^) was analyzed over three successive days, and the RSD values <4% were attained. The outcomes of the run–to–run precision and day-to-day precision data showed good precision for ultra-performance liquid chromatography-tandem mass spectrometry (UPLC-MS/MS) determination of acrylamide in food products.

A total of 56 food products of different brands and origin were studied, the samples include Arabic coffee *Qahwa*, coffee and tea. So as to overcome the sample matrix effects, the quantification of acrylamide in food samples was carried out using isotope dilution method utilizing AA-D_3_ as the labeled. The obtained results in terms of concentrations and recovery rates have been demonstrated in Tables 1, 2 and 3, where it can be observed that the acrylamide was identified in all of the analyzed food samples and concentrations reached up to 682 μg kg^−1^. The recovery rates were achieved between 86% and 93% in all studied food samples. The acrylamide concentrations in Arabic coffee *Qahwa* have been illustrated in Table 1. The acrylamide was detected in all samples in the range of 73 to 108 μg kg^−1^. The highest amount of acrylamide was identified in sample 7 (Maatouk) (108 μg kg^−1^), while sample 8 (Ajyal) contains the lowest level (73 μg kg^−1^). Apart from sample 7 (Maatouk), the other samples did not demonstrate a huge variation in acrylamide concentrations. Previously, a few studies relating to the determination of acrylamide in Arabic coffee samples have been reported^18^,^19^. In particular, one Arabic coffee (light roasted) sample from the Saudi Arabia^19^ and two samples including Arabic coffee and Arabic coffee (dark) from the Egypt^18^ have been studied. The obtained acrylamide concentrations are found totally different from our results. In the Arabic coffee (light roasted) sample from the Saudi Arabia, the author has not detected any amount of acrylamide^18^. Whereas, the studies performed in the Egypt, the author has detected acrylamide in both Arabic coffee (272 μg kg^−1^) and Arabic coffee (dark, 480 μg kg^−1^) samples^19^. The significant differences in the results might be due to the different analytical methodologies had applied or the samples had been thermally processed at different roasting temperatures which favor the acrylamide formation^20^.

Table 2 illustrates the amounts of acrylamide in roasted coffee of different brands and origin. The highest amounts of acrylamide were obtained in sample 1 (682 μg kg^−1^) whereas, sample 16 comparatively produced low amount (152 μg kg^−1^). This might have caused of high roasting temperature (160–210 °C) applied in sample 1 than sample 16. Formerly, a complete assessment on amounts of acrylamide in various food products has been reported^19^, particularly on coffee by the European Food Safety Authority^21^,^23^, and in few samples by Guenther *et al*. and others^22^,^23^,^24^. The levels of acrylamide reached up to 1047 μg kg^−1 ^24. In concurrence with the results obtained, the amounts of acrylamide in sample 2, sample 18, sample 19, sample 22 and sample 25 are found to be average values of 443 μg kg^−1^, which are similar to those reported by the Food and Drug Administration^25^. Alternatively, sample 1, sample 5, sample 6, sample 7 and sample 14 are found to be slightly higher concentrations with an average of 597 μg kg^−1^ and found in good agreements with the values obtained in the similar study^16^,^26^,^27^,^28^. These higher values are most likely as a result of the extreme roasting conditions and it is very difficult to compare with earlier published literatures. The concentrations of acrylamide in remaining samples including sample 3, sample 4, sample 8, sample 9, sample 10, sample 11–16, sample 17, sample 23 and sample 24 ranged from 152 to 368 μg kg^−1^. These values are in good agreement to those obtained in the European Union database^28^, where the lowest and highest concentrations oscillated from 79 to 2955 μg kg^−1^. Relatively, these low amounts, considering the existence of substantial precursors amount, are most likely due to the moderate roasting temperature applied in the preparation of these food products^29^.

About tea, a few studies relating to the occurrence of acrylamide are obtainable, thus it was very essential to make the screening of acrylamide contents in such kind of food samples. In the present study, a total of 22 tea samples of two classes (black and green) were studied, the obtained concentrations has been demonstrated in Table 3. The amounts of acrylamide in studied samples were found <100 μg kg^−1^. From the obtained data, it has been observed that the green types of tea samples contained low levels of acrylamide (10–18 μg kg^−1^), most likely the green tea were not dried out at elevated temperature. The black tea either loose or in bags comparatively comprise higher amounts of acrylamide ranged from 35 to 97 μg kg^−1^, this might be caused that these samples have been thermally processed at higher temperature between 100 °C and150 °C^29^. Our results are found in good agreement with those obtained in previous studies^29^,^30^ where the amounts of acrylamide in tea samples were also detected lower than 100 μg kg^−1^.

In comparison to coffee, the Arabic coffee *Qahwa* and tea contain lower amounts of acrylamide and below the consumption limit as recommended by the European Commission^31^. Among these hot drink samples, the Arabic coffee *Qahwa*, coffee and tea samples showed an important source of acrylamide and among these, tea especially green type contains lower amounts of acrylamide and can be considered as a heather drink followed by Arabic coffee *Qahwa* and coffee. Moreover, green coffee is also recognized for a variety of health advantages which linked with threat diminution of various chronic diseases, for instance cardiovascular, cancer and diabetes^32^.

**Figure 1:**
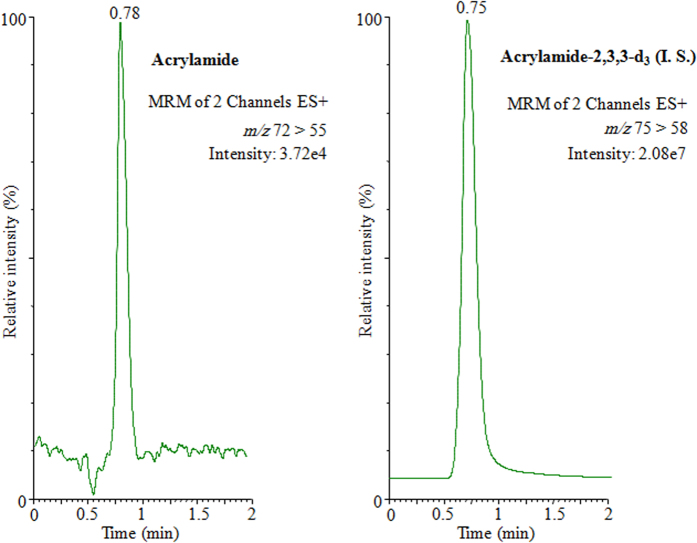
UPLC-MS/MS chromatograms of acrylamide and acrylamide-2,3,3-D_3_ (I. S.) in Arabic coffee *Qahwa* (sample 1, Harrari).

**Figure 2:**
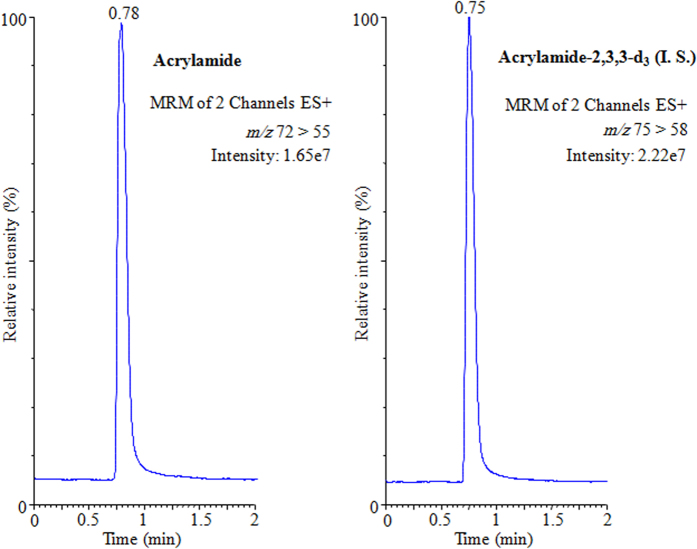
UPLC-MS/MS chromatograms of acrylamide and acrylamide-2,3,3-D_3_ (I. S.) in coffee (sample 1).

**Figure 3:**
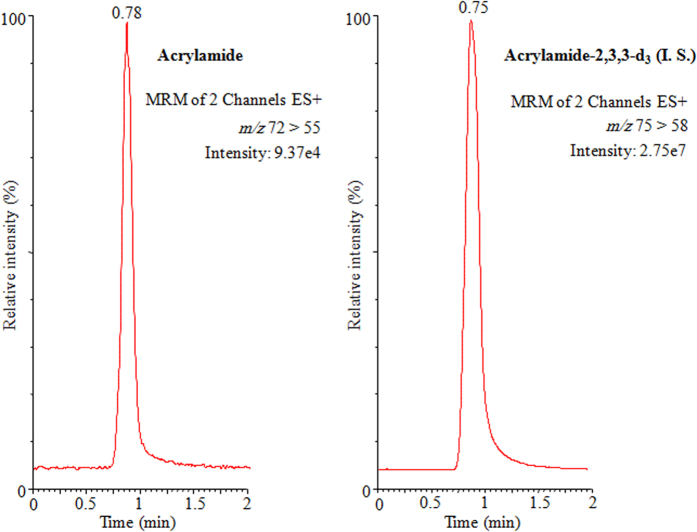
UPLC-MS/MS chromatograms of acrylamide and acrylamide-2,3,3-D_3_ (I. S.) in tea (sample 3, Black Fine).

To display the results, the UPLC-MS/MS acrylamide chromatograms of acrylamide in Arabic coffee *Qahwa* (sample 1, Harrari), coffee (sample 1) and tea (sample 3, Black Fine) have been illustrated in Figs 1, 2 and 3, respectively. The tremendous sensitivity, excellent peak symmetry and with no any interfering peaks arising at the elution time of target analyte was achieved.

## Conclusions

The contents of acrylamide in 56 food samples including Arabic coffee *Qahwa*, coffee and tea of different brands and origin were investigated. The acrylamide was detected in all of the analyzed samples and the concentrations ranged from 10 to 682 μg kg^−1^. In comparison to coffee, the Arabic coffee *Qahwa* and tea contain lower amounts of acrylamide. Additionally, the green tea contained low contents of acrylamide ranged 10 to 18 μg kg^−1^ and on the basis of acrylamide amounts we can assume green tea as a healthier hot drink. The excellent recovery rates (up to 93%) and quality parameters (linearity, LOD, LOQ and precisions) of the method were achieved. The outcomes revealed that the concentration of acrylamide in cooked foods show a big difference between diverse food groups and brands. This variance may be due to the initial concentration of amino acids especially asparagines and reducing sugars in food products, in addition to the roasting temperature and time, pH and water activity. The obtained data can also be used in epidemiological investigation to estimate the acrylamide exposure from nutritional survey.

## Materials and Methods

### Chemicals and reagents

**Figure 4:**
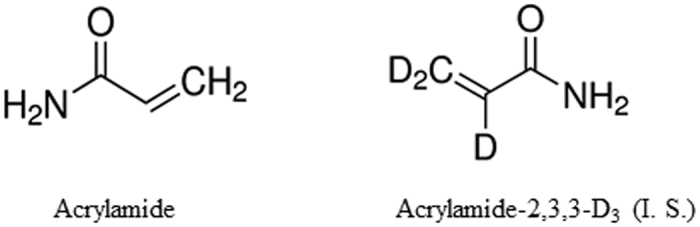
Structure of studied compounds.

Acrylamide (assay ≥ 99.8%), acrylamide–2,3,3–D_3_ (AA-D_3_, isotopic purity 98%) (Fig. 4), potassium hexacyanoferrate (assay ≥ 99.0%) and zinc acetate dihydrate (assay ≥ 98%) were purchased from Sigma–Aldrich (Sigma–Aldrich Chemie GmbH, Steinheim, Germany). Acetonitrile and methanol of HPLC–grade were purchased from Merck (Darmstadt, Germany). A Milli–Q water purification device, model advantage A10 from Millipore Corporation (Bedford, USA) was used to purify the water. Acrylamide stock solutions of 1 mg mL^−1^ and AA–D_3_ of 1 mg mL^−1^ were prepared with Milli–Q water. To get a series of standard solutions, the stocks solutions were diluted with Milli–Q water. Relating to the linearity, the calibration curve for the quantification of acrylamide in foods by UPLC-MS/MS was constructed and found linear over the range of 8–2000 μg L^−1^. All solutions were refrigerated at +4 °C until analysis. To get the Carrez I and II solutions, potassium hexacyanoferrate (10.6 g) and zinc acetate dihydrate (24 g) were individually dissolved in 100 mL of Milli–Q water.

Solid phase extraction (SPE) columns Strata™-X-C polymeric strong cation, 200 mg, 6 mL was obtained from Phenomenex (Torrance, USA) and Isolute^®^ ENV+, 200 mg, 3 mL was purchased from Biotage (Uppsala, Sweden). PTFE syringe filters (0.45 μm) were supplied from Macherey-Nagel GmbH (Düren, Germany). Nitrocellulose membrane, pore size 0.45 μm was purchased from Sigma-Aldrich (Sigma-Aldrich Chemie GmbH, Steinheim, Germany).

### Sample treatment

The cooked food samples (Arabic coffee *Qahwa*, coffee and tea) were purchased from a local supermarket (Riyadh, Saudi Arabia). The food samples were blended and homogenized by means of Microtron^®^ MB 800 (Kinematica AG, Littau, Switzerland), ultra-turrax T25 digital (IKA^®^-WERKE GmbH, Staufen, Germany) and coffee grinder CML-1000MKII (Stardust, Osaka, Japan), followed by passing through 250 μm sieve. Finally, the sieved samples were homogenized, bottled and labelled. Each bottle was packed with approximately 40 g of food sample and stored in a refrigerator at 4 °C until SPE procedures.

### Sample extraction method

Formerly ground and homogenized 2 g of subsamples were weighed into falcon tube (50 mL) followed by adding 10 mL water and 93 μL of internal standard AA–D_3_ (10 μg mL^−1^) into it. Falcon tubes containing sample were shaken for one hour on a tube rotator (Stuart, Staffordshire, United Kingdom). Subsequently, a HERMLE centrifuge, type Z32HK (Hermle Labortechnik GmbH, Wehingen, Germany) was used to centrifuge the sample tubes at 5000 rpm for 30 minutes. For the precipitation of co-extractives in the sample matrix, the clear sample supernatant was moved into another falcon tube and treated with 500 μL of Carrez I and II solutions, respectively. Subsequent centrifugation of the treated samples was performed at 5000 rpm for 3 min. Then, an aliquot (3 mL) of aqueous sample solution was filtered through a syringe filter (nitrocellulose membrane, pore size 0.45 μm) and loaded onto Strata™–X–C polymeric strong cation SPE column which was connected online with Visiprep™ vacuum manifold (Supelco, Gland, Switzerland). Subsequently, the Strata™–X–C column was eluted with water (3 mL) and the obtained eluent was loaded onto Isolute^®^ ENV + SPE column and eluted with a mixture of methanol:water (1 mL, 60:40, v/v). The sample extracts were gently evaporated to the final volume of 400 μL using Visidry™ vacuum manifold (Supelco, Gland, Switzerland) under nitrogen stream. Finally, the samples were filtered through PTFE syringe filters (0.45 μm) and moved into amber auto sampler glass vials for UPLC-MS/MS determination.

For the quantification of acrylamide in food samples, the isotope dilution method (a method of measuring the amount of chemical compounds in high matrix samples) has been employed. This method comprises the addition of known quantities of isotopically-enriched compound to the unknown analyzed samples, utilizing AA–D_3_ as the labeled compound was used to quantify the acrylamide in food samples. A response curve was constructed of area response ratio for *m/z* 55/58 (Table 4) versus the quantity of acrylamide injected with a constant quantity of [AA-D_3_]. This procedure offers more accurate results due to a correction of both extraction effectiveness and changes in the device response. At the beginning of the sample extraction procedure the known amount of AA–D_3_ standard solution was added to the food samples which allowed the quantification of the target compound^33^,^34^.

### Ultra-performance liquid chromatography-tandem mass spectrometry

The amount of acrylamide in food products was most frequently determined by liquid chromatography-tandem mass spectrometry (LC–MS/MS). Specifically, the LC–MS/MS has been considered a very compatible technique to the analysis of polar and non-volatile substances for instance acrylamide. In nature, the acrylamide is not sufficiently volatile and cannot be quantitatively analyzed by gas chromatography-mass spectrometry. Lately, an innovative technique based on UPLC-MS/MS has been applied for the analysis of acrylamide in food products. The UPLC-MS/MS technique offers rapid quantitative method in addition to excellent quality parameters for instance excellent linearity, precisions, selectivity and sensitivity^35^,^36^. For the determination of acrylamide in such types of highly matrixes samples, the use of triple quadrupole mass spectrometry system functioning in multiple reaction monitoring (MRM) mode is highly suggested due to its high selectiveness^36^. In the present study, the acrylamide in various food products were analyzed quantitatively determined by Waters Acquity^®^ UPLC method outfitted with a thermostat column compartment, quaternary pump, vacuum degasser and thermostat auto sampler (Milford, USA). The reversed phase Waters Acquity^®^ BEH C_18_ analytical column (50 mm × 2.1 mm i.d. and particle size, 1.7 μm) was used (Milford, USA) to get the separation of both acrylamide and AA-D_3_ analytes. The most favorable separation was attained in isocratic elution mode with mobile phase, methanol (10%) and 0.1% of formic acid in Milli–Q water (89.90%). The mobile phase flow rate and sample volume was 300 μL and 5 μL, correspondingly. In order to get the maximum instrumental sensitivity, at every ten samples injection, the BEH C_18_ analytical column was rinse with a mixture of methanol (50%) and water (50%).

The UPLC instrument was outfitted with ion source (electrospray ionization, ESI) and mass analyzer (Quattro Premier triple quadrupole) (Micromass, Milford, USA). To get the highest instrumental response relating to the determination acrylamide and AA-D_3_, the mass spectrometric system (MS) was functioned in positive ionization mode and data were attained MRM mode. The ESI source working parameters were as follows: Cone voltage, 48 V; capillary voltage, 3.5 kV; desolvation temperature, 350 °C; source temperature, 120 °C; desolvation gas flow rate, 600 L h^−1^; and cone gas flow rate, 60 L h^−1^. For cone and collision gases, nitrogen gas of high purity (99.99%), produced by a nitrogen generator (model NM30LA, Peak Scientific, Inchinnan, United Kingdom) and argon were used, correspondingly. To supply the primary vacuum to the MS instrument, an Oerlikon rotary pump (model SOGEVACSV40 BI, Cedex, France) was used. The MS/MS conditions for instance dwell times, collision energy voltages and the precursors and daughter ions associated to the chosen transitions of acrylamide and AA–D_3_, are demonstrated in Table 4. For the estimation of acrylamide and AA–D_3_, the most abundant daughter ions were chosen nevertheless, for the confirmation of acrylamide and AA–D_3_, the second-most abundant daughter ions were selected. The Waters MassLynx V4.1 software (Milford, USA) was used for the data acquisition^36^.

## Additional Information

**How to cite this article**: Khan, M. R. *et al*. Occurrence of acrylamide carcinogen in Arabic coffee *Qahwa*, coffee and tea from Saudi Arabian market. *Sci. Rep.* **7**, 41995; doi: 10.1038/srep41995 (2017).

**Publisher's note:** Springer Nature remains neutral with regard to jurisdictional claims in published maps and institutional affiliations.

## Figures and Tables

**Table 1:** Amount of acrylamide and recovery rates in cooked Arabic coffee *Qahwa*.

Sample	Type	Country of origin	Acrylamide (μg kg^−1^) ± SD	Recovery rates (%)
Sample 1	Harrari	Ethiopia	74 ± 3	89
Sample 2	Khaolani	Yemen	78 ± 3	90
Sample 3	Bareeah	Yemen	82 ± 4	92
Sample 4	Alasalah	Ethiopia	85 ± 4	90
Sample 5	Anoosh	—	79 ± 3	91
Sample 6	Bayouni	Turkey	80 ± 3	92
Sample 7	Maatouk	—	108 ± 6	90
Sample 8	Ajyal	—	73 ± 3	87
Sample 9	Yatooq	—	89 ± 4	92

—not described on label; SD = standard deviation.

**Table 2:** Amount of acrylamide and recovery rates in coffee products.

Sample*	Country of origin	Acrylamide (μg kg^−1^) ± SD	Recovery rates (%)
Sample 1	Brazil	682 ± 23	92
Sample 2	Brazil	412 ± 17	89
Sample 3	Switzerland	250 ± 12	90
Sample 4	Malaysia	246 ± 12	92
Sample 5	Ecuador	654 ± 22	92
Sample 6	Brazil	502 ± 20	90
Sample 7	Turkey	587 ± 22	92
Sample 8	Turkey	305 ± 14	93
Sample 9	Costa Rica	214 ± 10	87
Sample 10	Turkey	320 ± 15	90
Sample 11	Brazil	225 ± 14	88
Sample 12	Brazil	340 ± 15	86
Sample 13	Colombia	234 ± 12	91
Sample 14	Germany	560 ± 22	92
Sample 15	Switzerland	201 ± 10	91
Sample 16	Turkey	152 ± 8	88
Sample 17	Germany	247 ± 12	89
Sample 18	Germany	460 ± 18	93
Sample 19	Germany	475 ± 18	93
Sample 20	Germany	432 ± 17	91
Sample 21	Germany	360 ± 13	87
Sample 22	Netherlands	450 ± 18	91
Sample 23	France	368 ± 15	91
Sample 24	Switzerland	184 ± 10	90
Sample 25	Malaysia	430 ± 17	92

^*^All samples were ground coffee beans type except sample 4 and 17 (freeze dried); SD = standard deviation.

**Table 3:** Amount of acrylamide and recovery rates in tea products.

Sample	Type	Country of origin	Acrylamide (μg kg^−1^) ± SD	Recovery rates (%)
Sample 1	Black, Loose	—	84 ± 4	91
Sample 2	Black, Loose	Spain	73 ± 3	89
Sample 3	Black Fine, Loose	—	97 ± 6	92
Sample 4	Black, Loose	—	72 ± 3	88
Sample 5	Black, Loose	—	78 ± 4	90
Sample 6	Black, Loose	—	80 ± 5	88
Sample 7	Black, Loose	—	68 ± 3	91
Sample 8	Black, Bag	—	82 ± 4	92
Sample 9	Black, Bag	Kenya	74 ± 4	90
Sample 10	Black, Bag	UK	79 ± 4	92
Sample 11	Natural Green	—	18 ± 2	91
Sample 12	Green	—	23 ± 2	89
Sample 13	Pure Green	—	10 ± 1	90
Sample 14	Black, Bag	Sri Lanka	79 ± 4	91
Sample 15	Green	Sri Lanka	11 ± 1	90
Sample 16	Black, Loose	Kenya	74 ± 3	91
Sample 17	Black, Bag		72 ± 3	91
Sample 18	Earl Grey, Bag	UK	35 ± 2	88
Sample 19	Black, Bag	—	81 ± 4	91
Sample 20	Black, Bag	Sri Lanka	78 ± 4	92
Sample 21	Black, Bag	—	62 ± 3	92
Sample 22	Black, Bag		85 ± 4	89

—not described on label; SD = standard deviation; UK = United Kingdom.

**Table 4:** MRM conditions applied with the triple quadrupole mass spectrometric system*.

Compound	Precursor ion [M + H]^+^ (*m/z*)	Quantification		Confirmation	
		Product ion (*m/z*)	Collision energy (eV)	Product ion (*m/z*)	Collision energy (eV)
Acrylamide	72	55	48	44	43
Acrylamide-2,3,3-D_3_	75	58	52	47	47

^*^Dwell time (0.025 s) in both cases.
